# Association between health literacy and purpose in life and life satisfaction among health management specialists: a cross-sectional study

**DOI:** 10.1038/s41598-022-11838-w

**Published:** 2022-05-18

**Authors:** Nobutaka Hirooka, Takeru Kusano, Shunsuke Kinoshita, Ryutaro Aoyagi, Kohei Saito, Hidetomo Nakamoto

**Affiliations:** Department of General Internal Medicine, Saitama Medical University, Morohongo 38, Moroyama-cho, Iruma-gun, Saitama, 350-0495 Japan

**Keywords:** Psychology, Environmental social sciences

## Abstract

Purpose in life is anchored as a goal in national health promotion plans in several countries. Health literacy is the ability to find, understand, use, and evaluate health-related information; this ability has been investigated in terms of its effect on health outcomes and is said to play a critical role in health promotion. In the context of national health promotion, the effect of health literacy on purpose in life and life satisfaction remains unclear; therefore, this study aims to determine the effect of health literacy. A cross-sectional study was conducted on Japanese health management specialists (N = 1920). Health literacy was measured using the Communicative and Critical Health Literacy scale. Purpose in life and life satisfaction were measured using the Ikigai-9 scale and a Likert scale, respectively. We analyzed the associations between health literacy and purpose in life and life satisfaction with regression analyses. Age, sex, income, education, marital status, psychological stress, and diseases as present illness were adjusted in the statistical models. Our multiple linear regressions indicated that health literacy was significantly associated with purpose in life (*β* = 0.199, *p* < 0.001), after adjusting for covariates (age, sex, income, education, marital status, psychological factor, and disease status). Life satisfaction was also significantly associated with health literacy (*β* = 0.126, *p* < 0.001). Health literacy is associated with purpose in life and life satisfaction among specialists in health management. Health literacy plays a critical role in lifestyle-related disease prevention and health promotion. Interventions to improve health literacy may be warranted in the context of national health promotion.

## Introduction

The global population, especially that of Japan, is aging rapidly, and healthy aging is an interest that has been incorporated into national health promotion in many countries^1,2^. Having purpose in life is fundamental to human beings’ health and studies show that having purpose in life helps to overcome physical and psychological disorders including unhealthy behaviors^3–6^. Studies also showed that purpose in life reduces mortality from multiple diseases such as cardiovascular disease^7,8^, for which poor health-related behaviors are the risks. Ultimately, purpose in life improves health, and increases life-expectancy^8,9^. Thus, purpose in life is considered an important contributor to healthy aging. Purpose in life is defined as “a self-organizing life aim that stimulates goals^10^” and is known to promote healthy behaviors and give life meaning^10,11^. *Ikigai* is a Japanese word that is defined as something to live for, or the joy and goal of living^8,12^. *Ikigai* stands for what is considered to be an important factor for achieving better health and a fulfilling life^13^. Although *Ikigai* may not be fully comparable to purpose in life, it does include the concept and plays a cardinal role in yielding positive health-related outcomes^8^. Similarly, a growing number of studies show that life satisfaction is associated with better health outcomes^14–16^. People with higher life satisfaction engage in better health-related behaviors^17–19^. A lower prevalence of health behavior-related diseases, such as cardiovascular disease, has also been recorded among people with higher life satisfaction^20,21^. Therefore, both purpose in life and life satisfaction may be important methods for enhancing health and for successful aging. Because of the benefits of purpose in life and life satisfaction on health shown by many studies, both are expected to play a critical role in health policy^22,23^.

To fulfill these goals, governments in the United States, Europe, Australia, and China, among others, have developed national strategies to improve health literacy to promote health^24–26^. Japan has one of the most rapidly aging populations in the world and faces an increase in lifestyle-related diseases, health care costs, and the burden of nursing care for older adults^27^. Thus, a national health promotion program, Health Japan 21st Century (HJ21)^22,27,28^, and the Smart Life Project considered possible lifestyle-related disease prevention and health promotion by improving health literacy^29^. The ultimate goals of these programs are to provide citizens with opportunities to live healthy and meaningful lives, while also decreasing the societal and monetary burden of healthcare in a rapidly aging society.

The goals of national health promotions such as HJ21 include preventing diseases, reducing mortality, and increasing healthy longevity, and they also aim to improve social outcomes, such as quality of life, fulfillment in life, and satisfaction^22,27^. Nutbeam proposed a model of health promotion that includes both health and social outcomes as the ultimate goal^30^. In this model, health literacy plays a key role. Several studies have investigated the association between health literacy and social outcomes^31^; however, the results were inconsistent. In addition, social outcomes were defined in multiple ways, based on each study’s objectives. In that, purpose in life and life satisfaction are part of social outcome.

Health literacy is the ability to find, understand, evaluate, and use health-related information^32^. Health literacy has been recognized as an important determinant of health, and studies show that higher health literacy is associated with better health outcomes and positive health-related behaviors^33^. In addition, low health literacy is a predictor of poor health, both physically and mentally^34,35^, in multiple clinical settings^36,37^. Health literacy can be improved through education, personal forms of communication, and community-based educational outreach and with better information services^38,39^. In the health promotion context, health literacy enhances the capacity to influence social interaction and improves community empowerment^30^. Thus, health literacy can play a key role in national health promotion. Several reviews have pointed out problems in health literacy studies on health outcomes in clinical settings, including the quality of studies and a lack of standard measurements. They also highlighted the issue of a narrow range of health outcomes as well as the targeted group of patients. Therefore, there are considerable gaps in research addressing the effect of health literacy on health in the context of public health and health promotion in large populations.

There are also few studies that have investigated the association between health literacy and the specific concepts of purpose in life and life satisfaction from the perspective of national health promotion^40,41^. Investigating the effect of health literacy on purpose in life and life satisfaction will enable us to better understand the mechanistic link between health literacy and these social outcomes, and will assist in preparing for an effective intervention in health promotion practice.

The relationships between health literacy and purpose in life and life satisfaction in the context of national health promotion campaigns such as HJ21 and the Smart Life project in Japan, remains unclear. It is of great scientific value to investigate on the population which have gained health literacy in line with HJ21 if they increase purpose in life through gaining health literacy. The study target, health management specialists, selected in this study who have specific training to increase the capacity of health literacy and do not require any prior health-related degree or certificate is in line with the HJ21. Understanding these relationships will be insightful when planning specific health promotion interventions in national health campaigns. The study aims to investigate the extent to which purpose in life and life satisfaction are influenced by improving health literacy in the Japanese population.

## Methods

### Study design

The study design was a cross-sectional survey among professional health management specialists, part of an ongoing prospective cohort study. All questionnaires were sent with the study explanation and informed consent form. After reading the study explanation, the instruction indicated to sign the informed consent form to participate in the study. Then, questionnaires were administered when the study participants were enrolled. This study was approved by the Ethics Committee of Saitama Medical University (ID 926, 2020).

### Study participants

Study participants were health management specialists certified by the Japanese Association of Preventive Medicine for Adult Disease (JAPA)^42^. This certificate program for health management specialists is sponsored by the Japanese Ministry of Education, Culture, Sports, Science and Technology. These specialists are expected to conduct health promotion workshops and to engage the community they live in. Health management specialists are certified in multiple courses of study, with candidates studying various aspects within the course, including health promotion, lifestyle-related diseases, mental health, nutrition, environment and health, physical activity and exercise, emergency medicine, life support, and the health care system. To register, candidates must pass a final written examination.

The study population was identified as all individuals who are certified specialists in health management obtained from the register of the JAPA. Specialists were contacted to participate in the study by mail, distributed on August 1, 2020. The inclusion criteria of the study were continuing participation in professional education provided by the JAPA, and provision of written informed consent. We excluded specialists who did not engage in continuing education or health promotion activities. Among the individuals who met the inclusion criteria (*N* = 4530), a total of 1920 (response rate: 42.4%) agreed to participate in the prospective cohort study. All the individuals who agreed to participate in the cohort study were included in the cross-sectional analysis.

### Variables and measurements

Variables included demographic data (age, sex, education, marital status, family members living with, income, psychological stress, and disease status), health literacy, purposeful life score, and life satisfaction. Demographic data were measured with self-administered questionnaires. The item for the disease status included presence or absence of diabetes mellitus, hypertension, dyslipidemia, cancer, and obesity such as “Do you have diabetes mellitus?” with the answer “Yes” or “No”. The psychological stress was evaluated with 4-Likert scale; none, low, moderate, and high. Health literacy was measured using a formerly validated tool, the communicative and critical health literacy (CCHL) scale^43^. The CCHL measures a broader concept of health literacy defined as communicative and critical health literacy including the ability to extract, understand, and utilize health-related information. The items in the scale include questions on the respondent’s ability to (1) collect health-related information from various sources, (2) extract the information they want, (3) understand and communicate the obtained information, (4) consider the credibility of the information, and (5) make decisions based on the information. Each item was rated on a 5-point Likert-type scale, ranging from 1, “strongly disagree,” to 5, “strongly agree”^43^. Purpose in life was measured using a formerly validated tool, the Ikigai-9^44^. This tool consists of nine questions on various life purposes, with each question’s response ranging from 1, “strongly disagree,” to 5, “strongly agree.” The total score of the Ikigai-9 was calculated by adding the scores of all nine questions. Life satisfaction was also measured on a 5-point Likert-type scale with 5, “extremely satisfied”; 4, “satisfied”; 3, “neither satisfied nor dissatisfied”; 2, “dissatisfied”; and 1, “extremely dissatisfied.”

### Analysis

Descriptive statistics (mean, standard deviation, and range) were used to describe the characteristics of the study participants. Cronbach’s alpha of health literacy (CCHL) and Ikigai-9 were calculated to evaluate the inter-relatedness of the items for each measurement. Multiple linear regression analyses were performed to assess the purpose in life score and the life satisfaction score explained by health literacy. Three different models were fit to adjust for covariates. The first model included age, sex, income, education, and marital status as covariates. The second model included psychological stress as a covariate in addition to those included in the first model. The last model included disease status as an additional covariate. We considered *p* < 0.05 to be statistically significant. All statistical tests were two-tailed. IBM SPSS Statistics (Version 26.0. Armonk, NY, USA) software was used for the analyses.

### Ethics approval and consent to participate

This study compiled with all the principles of the Declaration of Helsinki and obtained approval from the university ethics board. Informed consent was obtained from all individual participants included in this study. The ethics committee of the Saitama Medical University approved the study (ID: 926, 2020).

## Results

Table 1 shows the demographic characteristics of the study participants. Overall, 1920 certified health management specialists were included in the study. More female specialists (*N* = 1181; 61.5%) participated in the study than males. Among the study participants, 73.9% of them were married. The ages of the study participants ranged from 22.0 to 93.0 years. More than 70% of the study participants had a higher education level than high school. The average and standard deviation of the CCHL score was 18.6 (95% CI [18.4–18.7]) and 3.6, respectively. The mean Ikigai-9 score was 33.8 (*SD* = 6.0, 95% CI [33.5–34.1]). The average and standard deviation for life satisfaction were 3.9 (95% CI [3.89–3.97]) and 0.8, respectively. Both Ikigai-9 and life satisfaction were normally distributed, assured by the histogram and the *P*–*P* plot of the score. The Cronbach’s α coefficient of the CCHL and Ikigai-9 were 0.862 and 0.842 respectively.

**Table 1:** Demographic characteristics of the cohort.

Characteristics	Total (*N* = 1920)
**Sex; *n* (%)**	
Male	739 (38.5)
Female	1181 (61.5)
**Age range; *n* (%)**	
< 30 years	24 (1.3)
30–39 years	109 (5.7)
40–49 years	312 (16.3)
50–59 years	596 (31.0)
60–69 years	552 (28.8)
70–79 years	277 (14.4)
≥ 80 years	50 (2.6)
**Age in years; mean (*SD*)**	58.5 (21.5)
**Education; *n* (%)**	
Junior high school	27 (1.4)
High school	511 (26.6)
Professional training college	331 (17.2)
College	296 (15.4)
University/Graduate school	755 (39.3)
**Marital status; *n* (%)**	
Married	1419 (73.9)
**Family; *n* (%)**	
Yes	1585 (82.6)
**Income (million Yen/year); *n* (%)**	
< 200	180 (9.4)
200–600	1017 (53.0)
> 600	717 (37.3)
Psychological stress score; mean (SD)	2.1 (0.76)
Cardiovascular disease or its risk factors; *n* (%)	834 (43.4)
Health literacy (CCHL); mean (SD)	18.6 (3.6)

*SD* standard deviation, *CCHL* the communicative and critical health literacy.

The multiple linear regressions indicated that health literacy was significantly associated with purpose in life after adjusting for covariates (age, sex, income, education, marital status, psychological stress, and disease status). The statistics for all models are shown in Table 2. Health literacy was the most important variable explaining the variability of life purpose in all models. Further, health literacy was significantly associated with purpose in life after adjusting for psychological stress (Model 2) and disease status (Model 3).

**Table 2:** Multiple regression analyses results of health literacy and purpose in life.

	*B* (SE)	*β*	*P*	*R*	Semi-partial *R*
Crude	0.402 (0.036)	0.247	< 0.001	0.25	–
Model 1	0.373 (0.035)	0.229	< 0.001	0.34	0.227
Model 2	0.333 (0.034)	0.204	< 0.001	0.44	0.202
Model 3	0.324 (0.034)	0.199	< 0.001	0.44	0.203

SE, standard error; *B,* unstandardized regression coefficients; *β*, standardized regression coefficients, *R*: coefficient of multiple regression, semi-partial *R*: semi-partial coefficient of health literacy in the multiple regression models.

Model 1 adjusted for age, sex, income, education, and marital status; Model 2, psychological stress; Model 3, status of diabetes mellitus, hypertension, dyslipidemia, cancer, and obesity, respectively.

Our multiple linear regressions also indicated that health literacy was statistically and significantly associated with life satisfaction after adjusting for covariates (age, sex, income, education, marital status, psychological factors, and disease status). The statistics for all models are shown in Table 3. Similar to the regression analysis between health literacy and purpose in life, health literacy was significantly associated with life satisfaction, after adjusting for psychological factors (psychological stress) and explained most of life satisfaction.

**Table 3:** Multiple regression analyses results of health literacy and life satisfaction.

	*B* (SE)	*β*	*P*	*R*	Semi-partial R
Crude	0.031 (0.005)	0.139	< 0.001	0.14	–
Model 1	0.030 (0.005)	0.134	< 0.001	0.18	0.132
Model 2	0.028 (0.005)	0.125	< 0.001	0.21	0.123
Model 3	0.029 (0.005)	0.126	< 0.001	0.21	0.124

SE, standard error; *B,* unstandardized regression coefficients; *β*, standardized regression coefficients, *R*: coefficient of multiple regression, semi-partial *R*: semi-partial coefficient of health literacy in the multiple regression models.

Model 1 adjusted for age, sex, income, education, and marital status; Model 2, psychological stress; Model 3, status of diabetes mellitus, hypertension, dyslipidemia, cancer, and obesity, respectively.

## Discussion

To the best our knowledge, this is the first study to show that health literacy is associated with both purpose in life and life satisfaction, among individuals with a wide age range and in a nonclinical setting. Musich et al. showed a positive association between health literacy and purpose in life among older adults^33^. Their study used a single question to measure health literacy. In our study, the results were confirmed using a validated tool to measure health literacy. Thus, our study results expand the existing evidence to individuals of a wider age range.

Many studies show that purpose in life and life satisfaction decrease the risk of premature mortality and improve health outcomes^5,7,45,46^. Moreover, an enhanced purpose in life seems to result in better health outcomes^33,47^. Previous Japanese studies show an association between purpose in life and all-cause mortality, as well as cause-specific mortality, such as cardiovascular diseases^48^. Similarly, life satisfaction has also been investigated in relation to health behaviors and health outcomes including chronic diseases such as diabetes, cancer, cardiovascular diseases, and mortality^49–55^. Our study supports the existing evidence that health literacy influence both purpose in life and life satisfaction in the context of health promotion.

The mechanistic link between purpose in life and life satisfaction and health outcomes have been hypothesized in several ways. Kim et al. suggested three key biobehavioral pathways by which a sense of purpose in life reduce the risk of cardiovascular disease: (1) psychological and stress-buffering, (2) behavioral pathways, and (3) biological pathways^56^. Previous studies also show a positive association between health literacy and health-related lifestyle behavior^57,58^. A noteworthy highlight in our study results is the positive association between health literacy and purpose in life and life satisfaction. Health literacy includes the capacity to make better health decisions and was shown to be associated with higher sense of purpose in life and life satisfaction in this study. Based on the association of health literacy with an increased sense of purpose in life and life satisfaction, increasing health literacy might causally prevent diseases and decrease mortality through purpose in life^59^. Therefore, it is reasonable to assume that a higher sense of purpose in life due to health literacy interventions may have a causal influence on health promotion and disease prevention. To understand the mechanism of the association between health literacy, purpose in life and life satisfaction, and how health literacy interventions can improve health outcomes through a higher sense of purpose in life and life satisfaction, a longitudinal study would be of great value. The mechanism may provide important insights when planning national health promotion.

The results of our study provide important insights for national health promotion planning and related policy making. The results support that increasing health literacy may improve citizens’ purpose in life. In Japan, extending health and longevity is included in the action plan, which is the platform for national health promotion called the health longevity plan^23^. The theoretical background for this national policy is to extend individuals’ health longevity through multiple interventions, such as lifestyle modification, social engagement, and disease prevention, while maintaining their quality of life and purpose in life^22,23^. Health literacy has been shown to be associated with a healthy lifestyle and ultimately, disease prevention^34–38^. Our study shows the potential benefit for purpose in life through increasing health literacy. Therefore, purpose in life may be a realistic target to help achieve the final goal, disease prevention and high purpose in life, through health literacy education. To improve the effect of health literacy, a longitudinal study is needed to investigate the causal mechanism of health literacy leading to good health outcomes and health-related goals.

Our study has several strengths. First, the participants’ age ranged from 22 to 93 years. It can therefore be said that the positive association between health literacy and purpose in life and life satisfaction among participants with an age range this wide is meaningful in the context of national health promotion. This is particularly important in health literacy research, as there is a lack of life-course evidence of health literacy on health outcomes. Second, this is the first study to support the theoretical base of the national health promotion activity in Japan. At a national level, this study provides evidence for a promising action plan.

There are also several limitations to the study. First, measurements including life satisfaction were based on self-administered questionnaires, which may produce measurement bias. Life satisfaction was measured using a simple Likert scale developed by researchers in the study, therefore it may need alternative measures that are valid across a broader range of the populations. Objectively measured health literacy would strengthen the study; however, no methods to objectively measure health literacy in a broad public health understanding is currently available. Second, 42.4% of the individuals who met the inclusion criteria participated in the study. This is a low response rate and indicates that selection bias might be present. Third, a cross-sectional design makes it impossible to determine the causality between health literacy and purpose in life and life satisfaction. How health literacy influences purpose in life or life satisfaction requires a longitudinal study, which is of great scientific interest in the future. Lastly, the issue of generalizability of the study results remains since the study population was the group of health specialists. While the purpose of choosing this target population was because the research hypothesis is that gaining health literacy is associated with purpose in life and life satisfaction. The target population who gained health literacy through the process of certification and showed that higher purpose in life and life satisfaction supported the concept of the HJ21. However, further generalizability in applying our results should be investigated.

## Conclusion

Health literacy is associated with purpose in life and satisfaction among health management specialists. These results support the theoretical background of the current national effort to prevent lifestyle-related diseases and to promote health based on health literacy education. Interventions to improve health literacy may be warranted in the context of national health promotion to reach its goals.

## Data Availability

The datasets used in the current study are available from the corresponding author upon reasonable request.

## Abbreviations

HJ21: Health Japan 21st Century
CCHL: Communicative and Critical Health Literacy
